# Supplementary material for: Ventilatory Effects of Isoflurane Sedation via the Sedaconda ACD-S versus ACD-L: A Substudy of a Randomized Trial
Source: J Clin Med. 2023 May 6;12(9):3314. doi: 10.3390/jcm12093314 (PMC10179426; doi:10.3390/jcm12093314)
Supplement: Supplementary file 1 [file jcm-12-03314-s001.zip › jcm-2310865-supplementary.pdf]

Supplement:

Ventilatory effects of isoflurane sedation via the Sedaconda ACD-S versus ACD-L: a substudy of a randomized trial

Lukas M. Müller-Wirtz[1,2] MD, Tobias Becher[3] MD, Ulf Günther[4] MD, Martin Bellgardt[5] MD, Peter Sackey[6] MD, Thomas Volk[1,2] MD, Andreas Meiser[1] MD

1) Department of Anaesthesiology, Intensive Care and Pain Therapy, Saarland University Medical Center and Saarland University Faculty of Medicine, Homburg, Saarland, Germany (thomas.volk@uks.eu; andreas.meiser@uks.eu)

2) OUTCOMES RESEARCH Consortium, Cleveland, Ohio, USA

3) Department of Anesthesiology and Intensive Care Medicine, University Medical Center Schleswig-Holstein, Campus Kiel, Kiel, Germany (tobias.becher@uksh.de)

4) University Clinic of Anaesthesiology, Intensive Care, Emergency Medicine, Pain Therapy, Klinikum Oldenburg, Oldenburg, Germany (guenther.ulf@klinikum-oldenburg.de)

5) St. Josef - Hospital Bochum, Department of Anaesthesiology and Intensive Care Medicine, University Hospital of the Ruhr-University Bochum, Bochum, Germany (martin.bellgardt@kklbo.de)

6) Department of Physiology and Pharmacology, Unit of Anesthesiology and Intensive Care, Karolinska Institute, Stockholm, Sweden (peter.sackey@ki.se)

Corresponding author:

Lukas Martin Müller-Wirtz, MD
Department of Anaesthesiology, Intensive Care and Pain Therapy
Saarland University Medical Center and Saarland University Faculty of Medicine
66421 Homburg / Germany
Phone: +49 (0) 6841 16 22485
Email: lukas.mueller-wirtz@uks.eu
ORCID: 0000-0002-7984-1798

Table S1: Inclusion and exclusion criteria

**Inclusion criteria**	**Exclusion criteria**
Patients ≥18 years	Never at target RASS in 8 hours prior to randomization
Continuous invasive ventilation and sedation ≤48 hours at start of study sedation	History of or genetic predisposition to malignant hyperthermia
Clinically likely to need invasive ventilation and sedation ≥24 hours at randomization	Uncompensated circulatory failure (MAP <55 mmHg despite IV fluids and vasopressors)
Ongoing sedation with propofol at time of randomization	Severe hepatic impairment (Child–Pugh Score C)
Prescribed target sedation depth within the RASS range –1 to –4	Significant laboratory abnormalities
Signed informed consent, or emergency inclusion criteria fulfilled and documented	Acute neuropathology without ICP monitoring (including but not limited to stroke, neurosurgery, and head trauma)
	Scheduled for surgery within 24 hours from randomization
	Tidal volume <350 mL
	History of any clinically significant disease in the opinion of the Investigator
	Need for continuous muscle relaxation at the time of randomization
	Pregnancy
	History of allergy/hypersensitivity to isoflurane or propofol
	Known participation in any other clinical study that included drug treatment within 3 months of the first administration of study drug
	Documented limitation of medical treatment

ICP=intracranial pressure. IV=intravenous. MAP=mean arterial pressure. RASS=Richmond Agitation–Sedation Scale.

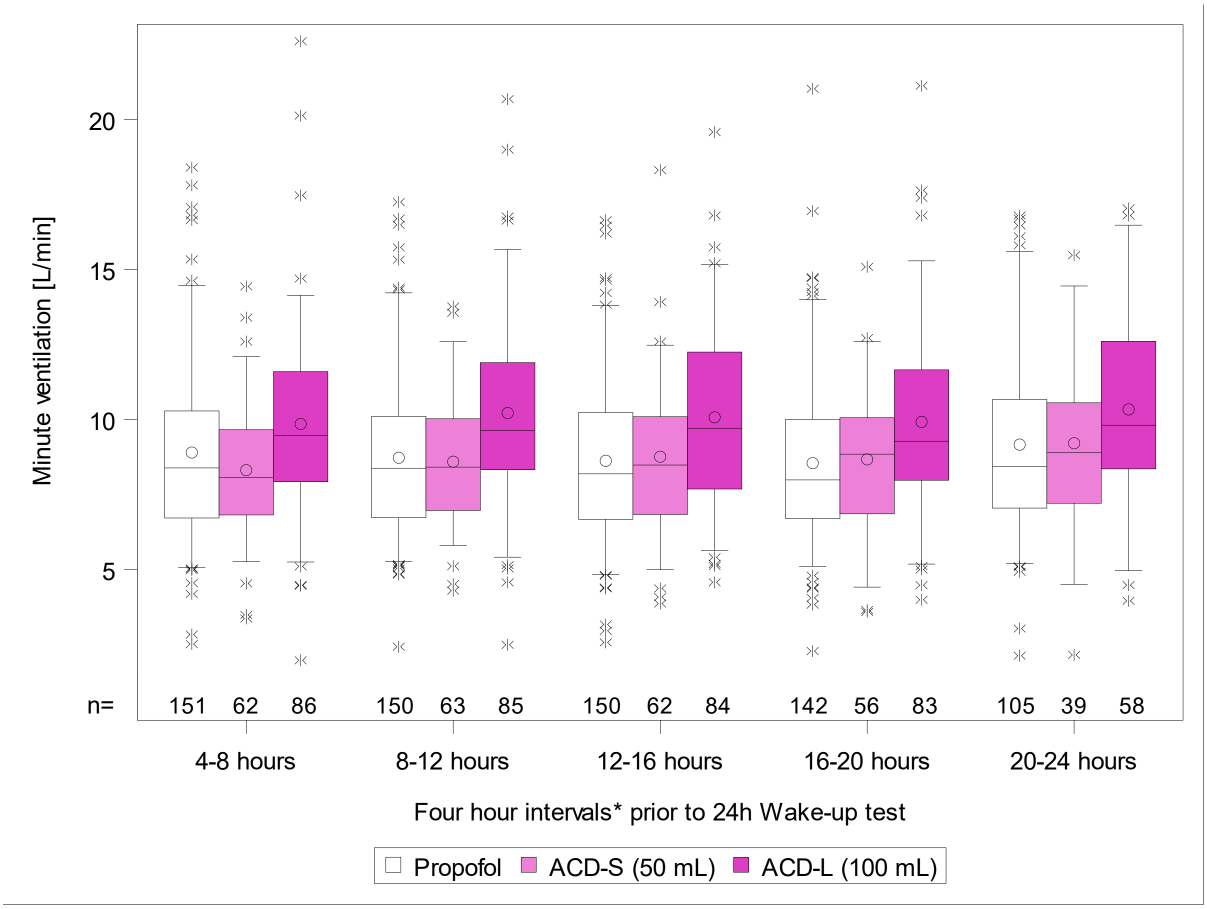

Figure S1: Minute ventilation

* Intervals 0-4 hours and 24-28 hours have been excluded from analysis, because of the limited number of assessments available. Boxes present means, medians, and interquartile ranges. Whiskers present lowest and highest observed value within 5th percentile low and the 95th percentile high. Stars represent outliers. ACD-S, anesthetic conserving device with 50 mL internal volume. ACD-L, anesthetic conserving device with 100 mL internal volume.

<u>Average differences [95% confidence interval]:</u>

ACD-L versus Propofol: 1.3 [0.7, 1.8]; p<0.001

ACD-S versus Propofol: -0.1 [-0.7, 0.6]; p=0.822

ACD-L versus ACD-S: 1.3 [0.6, 2.0]; p<0.001

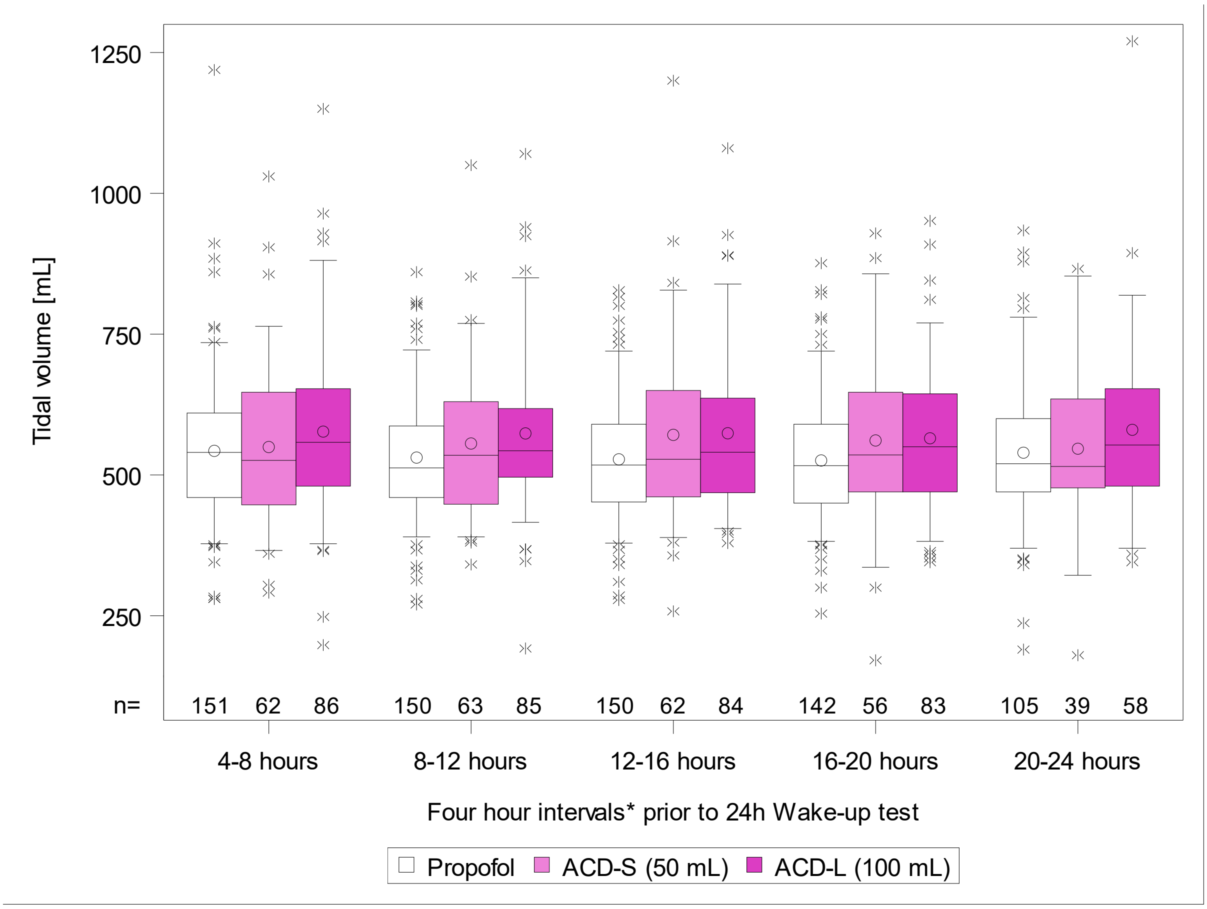

Figure S2: Tidal volume

* Intervals 0-4 hours and 24-28 hours have been excluded from analysis, because of the limited number of assessments available. Boxes present means, medians, and interquartile ranges. Whiskers present lowest and highest observed value within 5th percentile low and the 95th percentile high. Stars represent outliers. ACD-S, anesthetic conserving device with 50 mL internal volume. ACD-L, anesthetic conserving device with 100 mL internal volume.

<u>Average differences [95% confidence interval]:</u>

ACD-L versus Propofol: 44 [16, 72]; p=0.002

ACD-S versus Propofol: 24 [-7, 55]; p=0.126

ACD-L versus ACD-S: 20 [-15, 55]; p=0.255

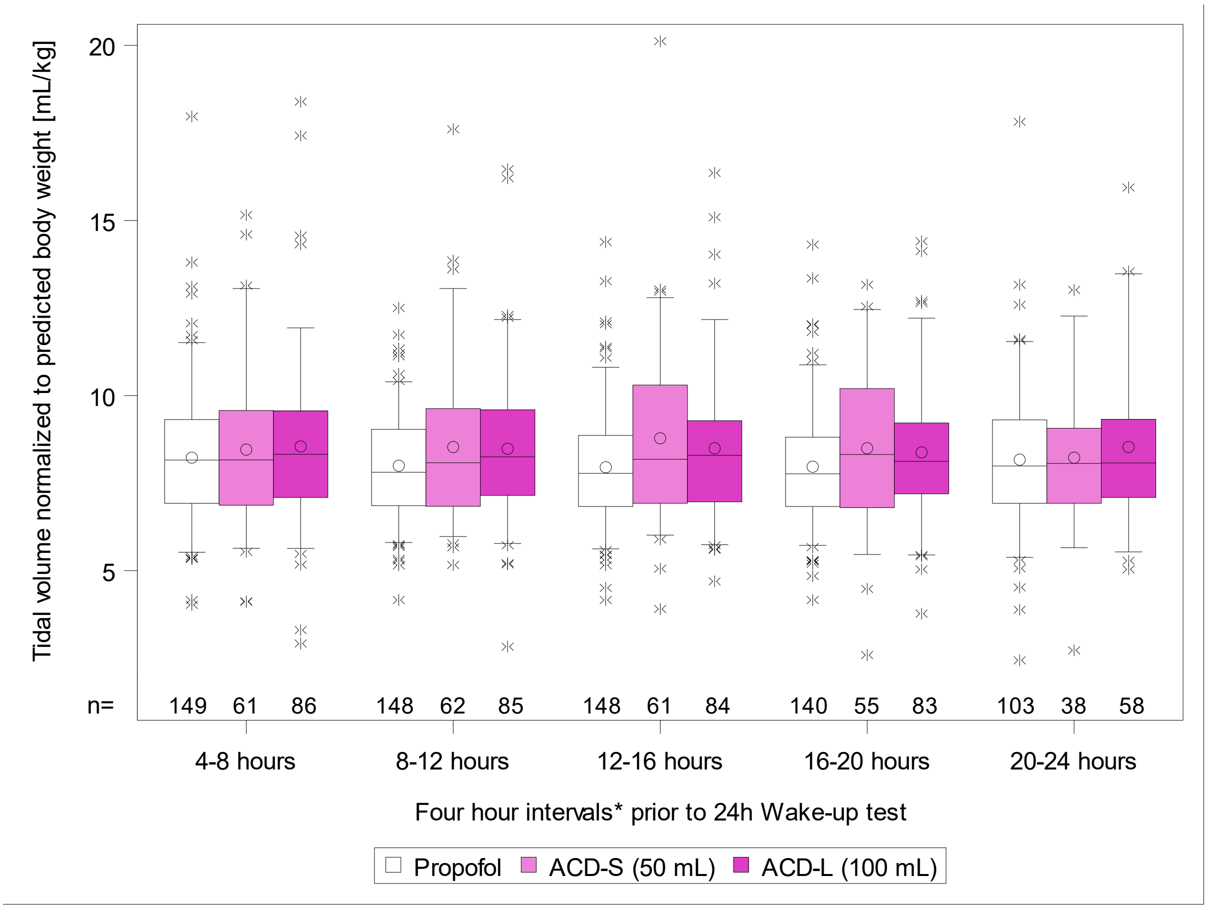

Figure S3: Tidal volume normalized to predicted body weight

* Intervals 0-4 hours and 24-28 hours have been excluded from analysis, because of the limited number of assessments available. Boxes present means, medians, and interquartile ranges. Whiskers present lowest and highest observed value within 5th percentile low and the 95th percentile high. Stars represent outliers. ACD-S, anesthetic conserving device with 50 mL internal volume. ACD-L, anesthetic conserving device with 100 mL internal volume.

<u>Average differences [95% confidence interval]:</u>

ACD-L versus Propofol: 0.45 [0.00, 0.90]; p=0.049

ACD-S versus Propofol: 0.47 [-0.03, 0.97]; p=0.067

ACD-L versus ACD-S: -0.02 [-0.58, 0.55]; p=0.951

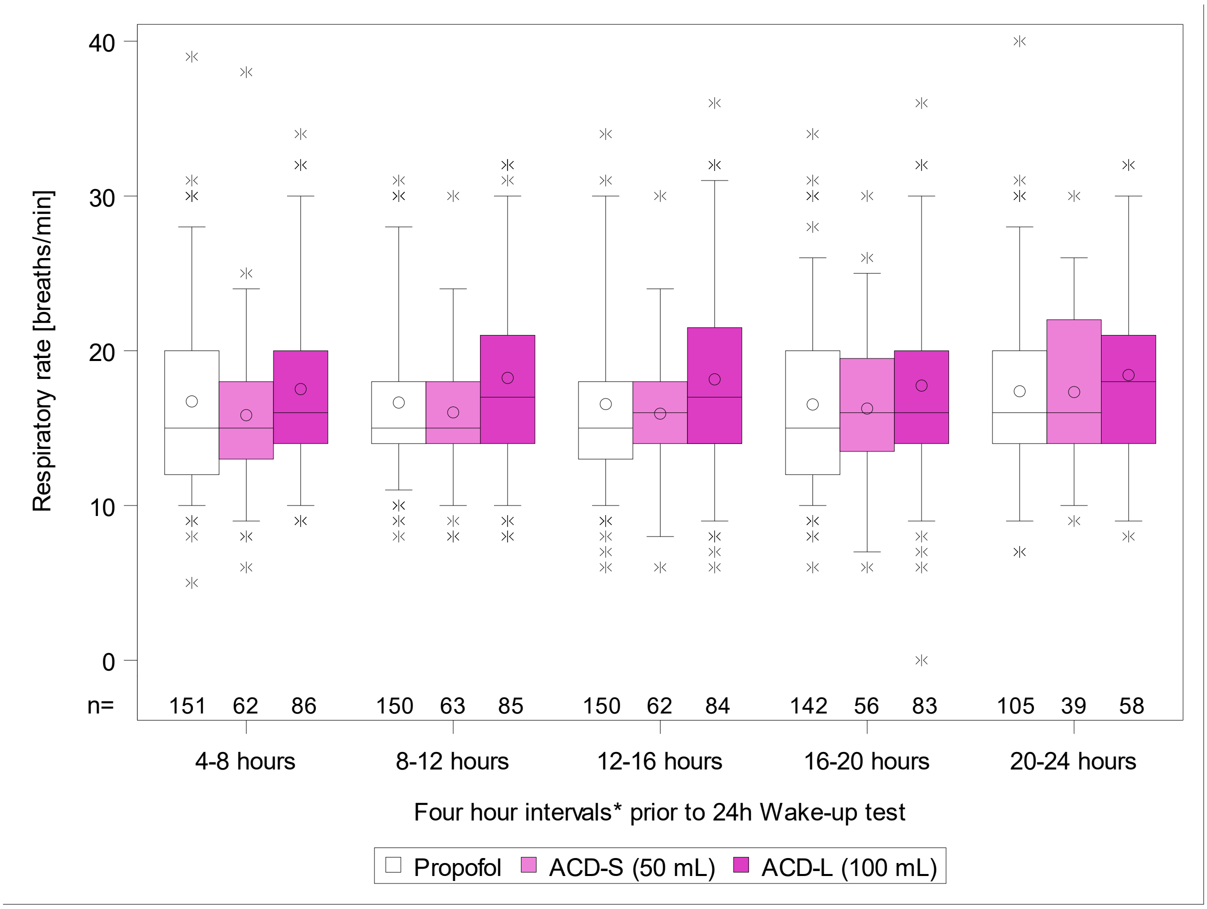

Figure S4: Respiratory rate

* Intervals 0-4 hours and 24-28 hours have been excluded from analysis, because of the limited number of assessments available. Boxes present means, medians, and interquartile ranges. Whiskers present lowest and highest observed value within 5th percentile low and the 95th percentile high. Stars represent outliers. ACD-S, anesthetic conserving device with 50 mL internal volume. ACD-L, anesthetic conserving device with 100 mL internal volume.

<u>Average differences [95% confidence interval]:</u>

ACD-L versus Propofol: 1.2 [0.1, 2.2]; p=0.025

ACD-S versus Propofol: -0.5 [-1.6, 0.6]; p=0.392

ACD-L versus ACD-S: 1.7 [0.4, 2.9]; p=0.011

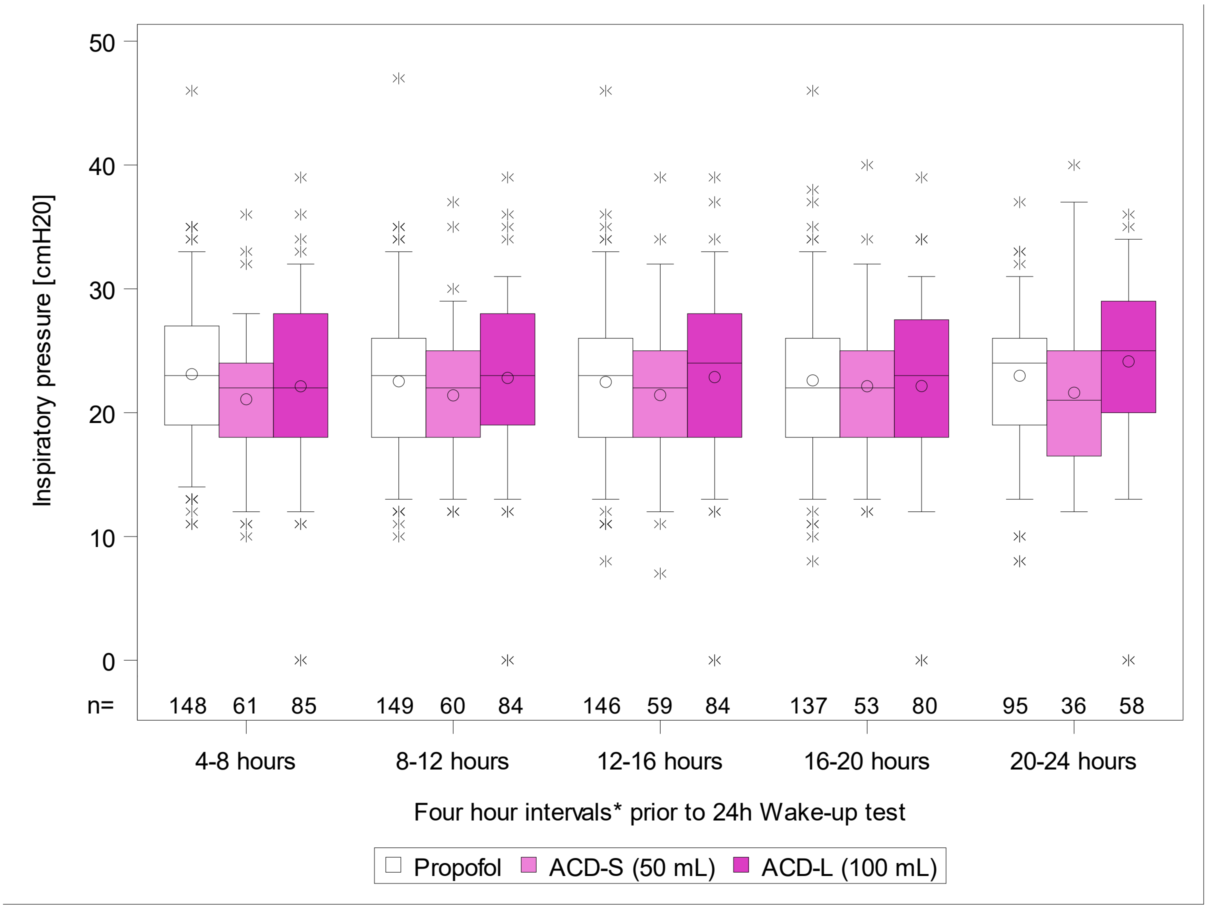

Figure S5: Inspiratory pressure

* Intervals 0-4 hours and 24-28 hours have been excluded from analysis, because of the limited number of assessments available. Boxes present means, medians, and interquartile ranges. Whiskers present lowest and highest observed value within 5th percentile low and the 95th percentile high. Stars represent outliers. ACD-S, anesthetic conserving device with 50 mL internal volume. ACD-L, anesthetic conserving device with 100 mL internal volume.

<u>Average differences [95% confidence interval]:</u>

ACD-L versus Propofol: 0.2 [-1.2, 1.6]; p=0.799

ACD-S versus Propofol: -1.3 [-3.0, 0.3] p=0.098

ACD-L versus ACD-S: 1.5 [-0.3, 3.3]; p=0.095

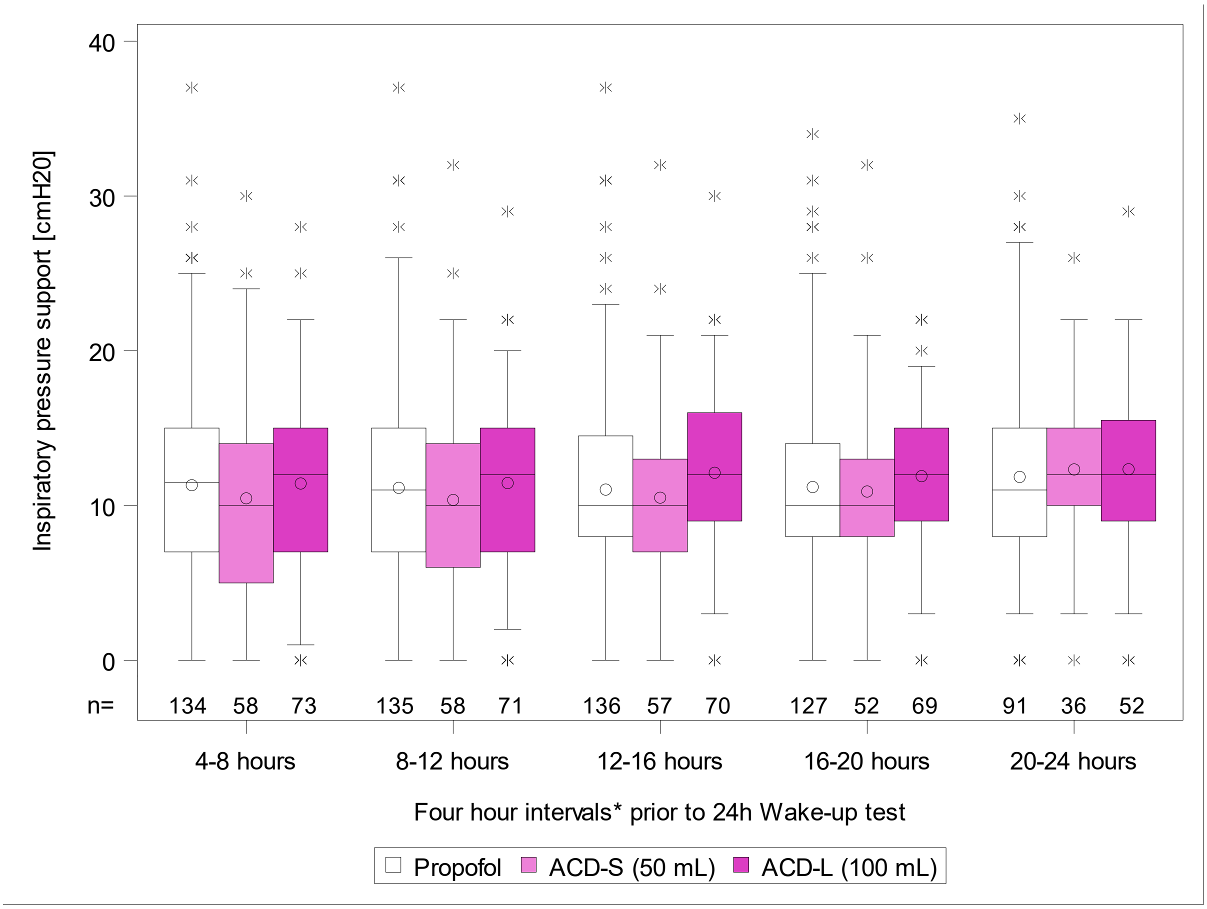

Figure S6: Inspiratory pressure support during spontaneous ventilation (pressure support above positive end-expiratory pressure)

* Intervals 0-4 hours and 24-28 hours have been excluded from analysis, because of §§the limited number of assessments available. Boxes present means, medians, and interquartile ranges. Whiskers present lowest and highest observed value within 5th percentile low and the 95th percentile high. Stars represent outliers. ACD-S, anesthetic conserving device with 50 mL internal volume. ACD-L, anesthetic conserving device with 100 mL internal volume.

<u>Average differences [95% confidence interval]:</u>

ACD-L versus Propofol: 0.6 [-0.8, 2.1]; p=0.393

ACD-S versus Propofol: -0.6 [-2.2, 1.0]; p=0.492

ACD-L versus ACD-S: 1.2 [-0.6, 3.0]; p=0.197

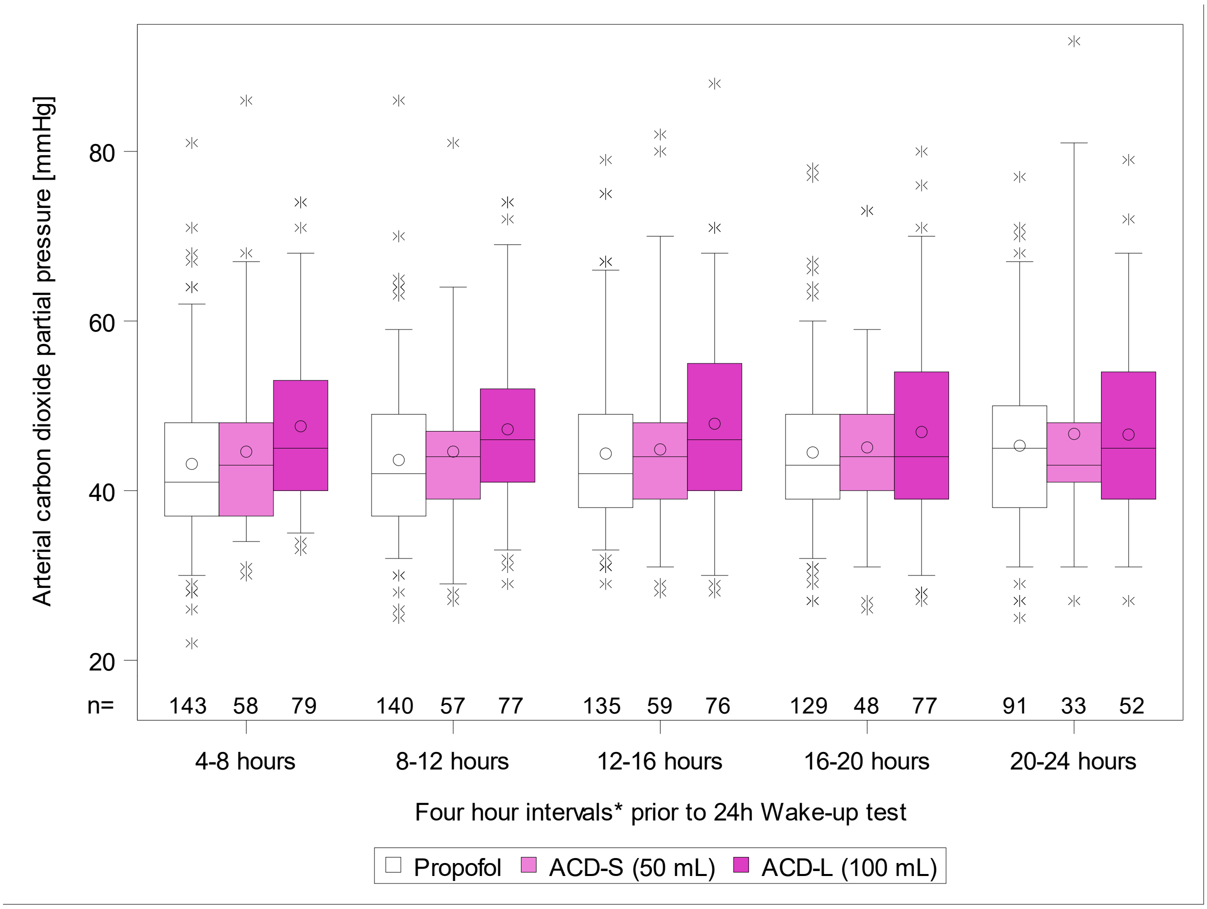

Figure S7: Arterial carbon dioxide partial pressure

* Intervals 0-4 hours and 24-28 hours have been excluded from analysis, because of the limited number of assessments available. Boxes present means, medians, and interquartile ranges. Whiskers present lowest and highest observed value within 5th percentile low and the 95th percentile high. Stars represent outliers. ACD-S, anesthetic conserving device with 50 mL internal volume. ACD-L, anesthetic conserving device with 100 mL internal volume.

<u>Average differences [95% confidence interval]:</u>

ACD-L versus Propofol: 3.4 [1.2, 5.6]; p=0.002

ACD-S versus Propofol: 1.1 [-1.4, 3.5]; p=0.380

ACD-L versus ACD-S: 2.3 [-0.4, 5.1]; p=0.098

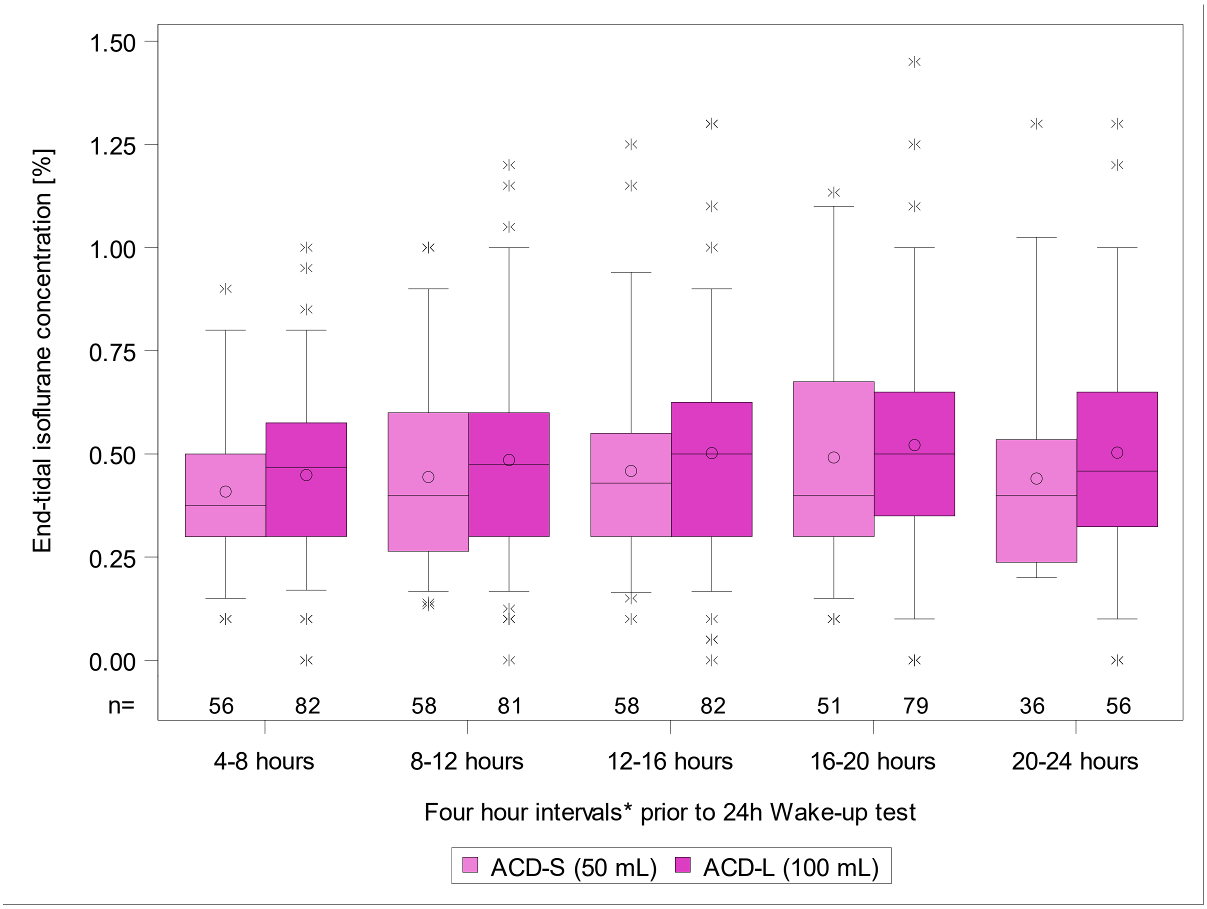

Figure S8: End-tidal isoflurane concentration

* Intervals 0-4 hours and 24-28 hours have been excluded from analysis, because of the limited number of assessments available. Boxes present means, medians, and interquartile ranges. Whiskers present lowest and highest observed value within 5th percentile low and the 95th percentile high. Stars represent outliers. ACD-S, anesthetic conserving device with 50 mL internal volume. ACD-L, anesthetic conserving device with 100 mL internal volume.

<u>Average differences [95% confidence interval]:</u>

ACD-L versus ACD-S: 0.04 [-0.03, 0.10]; p=0.287

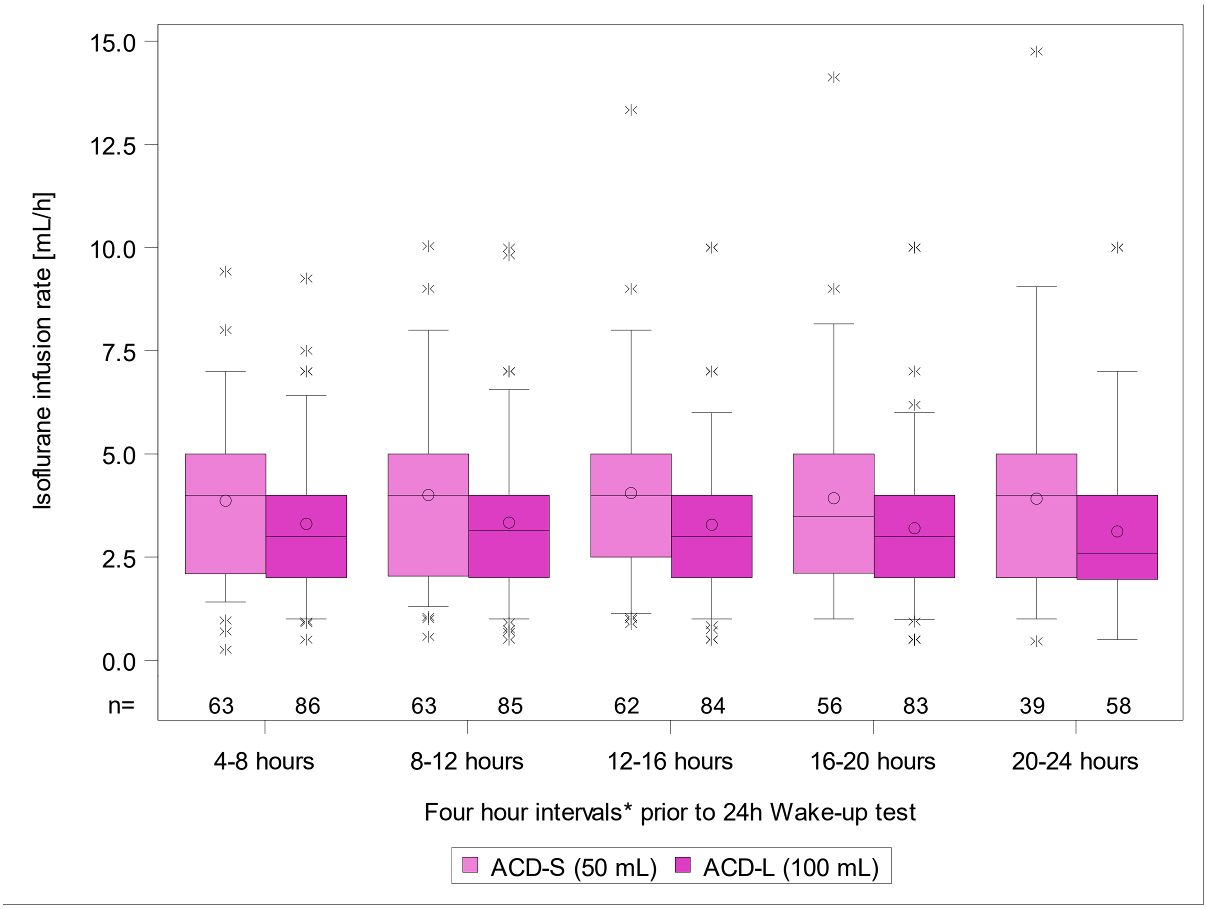

Figure S9: Isoflurane infusion rate

* Intervals 0-4 hours and 24-28 hours have been excluded from analysis, because of the limited number of assessments available. Boxes present means, medians, and interquartile ranges. Whiskers present lowest and highest observed value within 5th percentile low and the 95th percentile high. Stars represent outliers. ACD-S, anesthetic conserving device with 50 mL internal volume. ACD-L, anesthetic conserving device with 100 mL internal volume.

<u>Average differences [95% confidence interval]:</u>

ACD-L versus ACD-S: -0.7 [-1.3, 0.1] mL/h; p=0.022

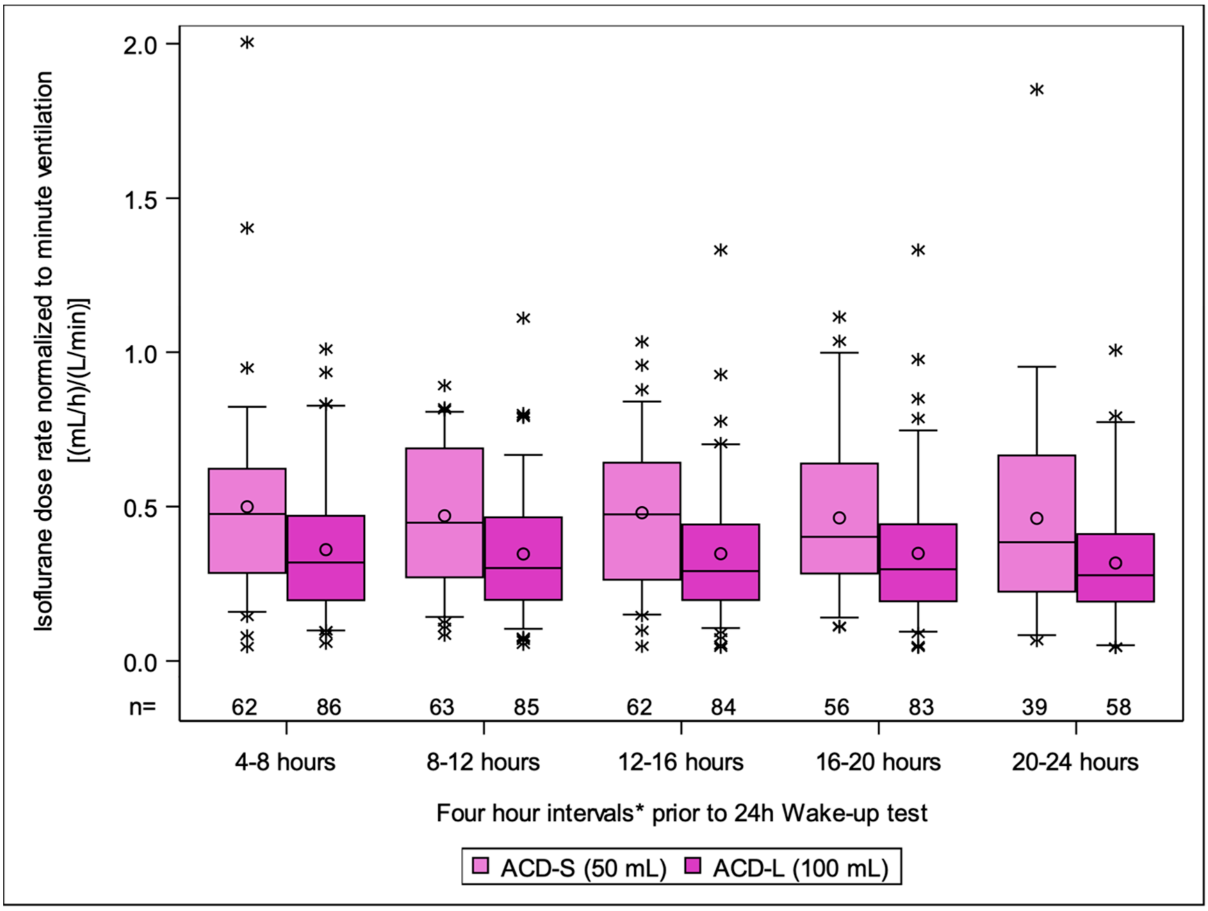

Figure S10: Isoflurane consumption normalized to minute ventilation

* Intervals 0-4 hours and 24-28 hours have been excluded from analysis, because of the limited number of assessments available. Boxes present means, medians, and interquartile ranges. Whiskers present lowest and highest observed value within 5th percentile low and the 95th percentile high. Stars represent outliers. ACD-S, anesthetic conserving device with 50 mL internal volume. ACD-L, anesthetic conserving device with 100 mL internal volume.

<u>Average differences [95% confidence interval]:</u>

ACD-L versus ACD-S: -0.13 [-0.20, -0.07]; $p<0.001$

Subgroup analysis: Chronic Obstructive Pulmonary Disease (COPD)

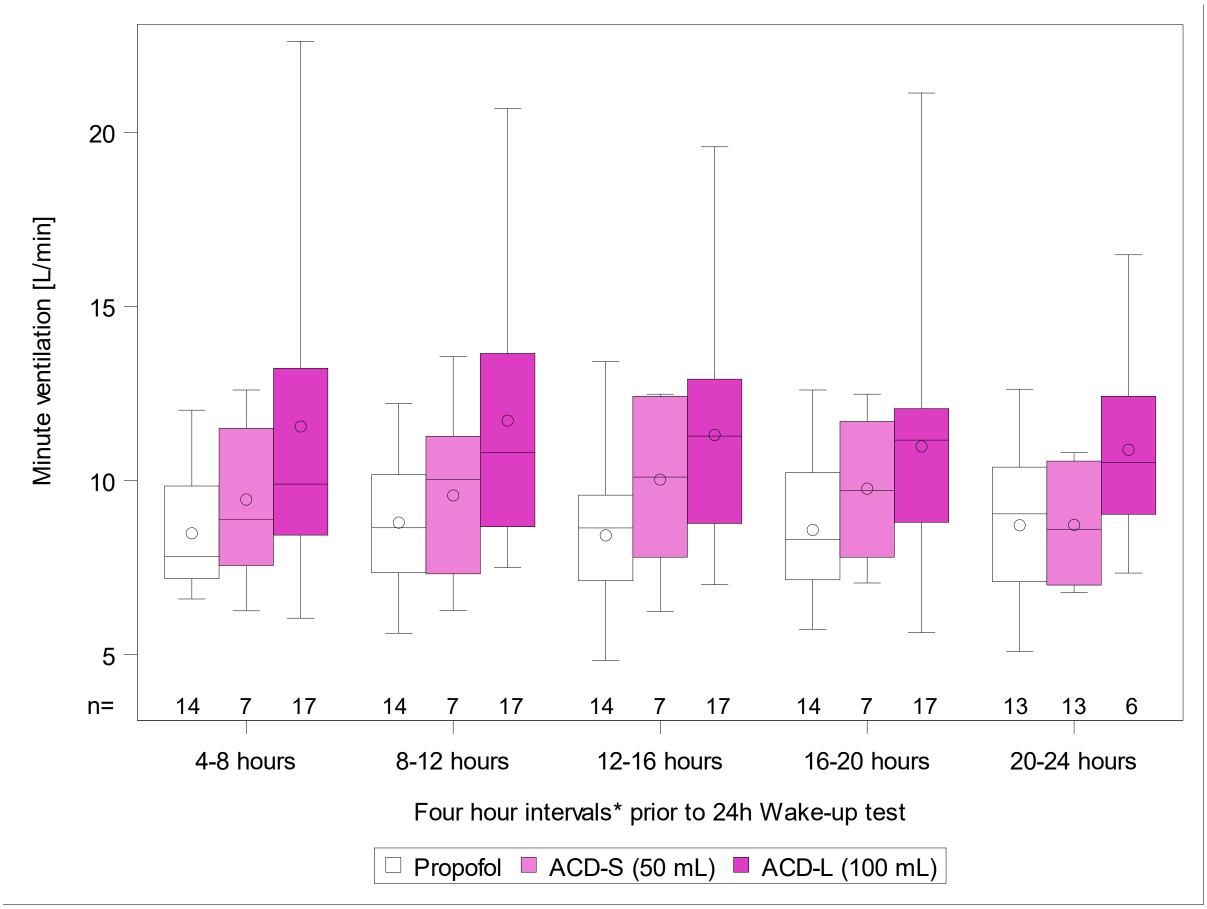

Figure S11: Minute ventilation in COPD patients

* Intervals 0-4 hours and 24-28 hours have been excluded from analysis, because of the limited number of assessments available. Boxes present means, medians, and interquartile ranges. Whiskers present lowest and highest observed value within 5th percentile low and the 95th percentile high. Stars represent outliers. ACD-S, anesthetic conserving device with 50 mL internal volume. ACD-L, anesthetic conserving device with 100 mL internal volume. COPD, chronic obstructive pulmonary disease.

<u>Average differences [95% confidence interval]:</u>

ACD-L versus Propofol: 2.0 [0.3, 3.7]; p=0.024

ACD-S versus Propofol: -0.8 [-3.2, 1.6]; p=0.511

ACD-L versus ACD-S: 2.8 [0.7, 4.9]; p=0.011

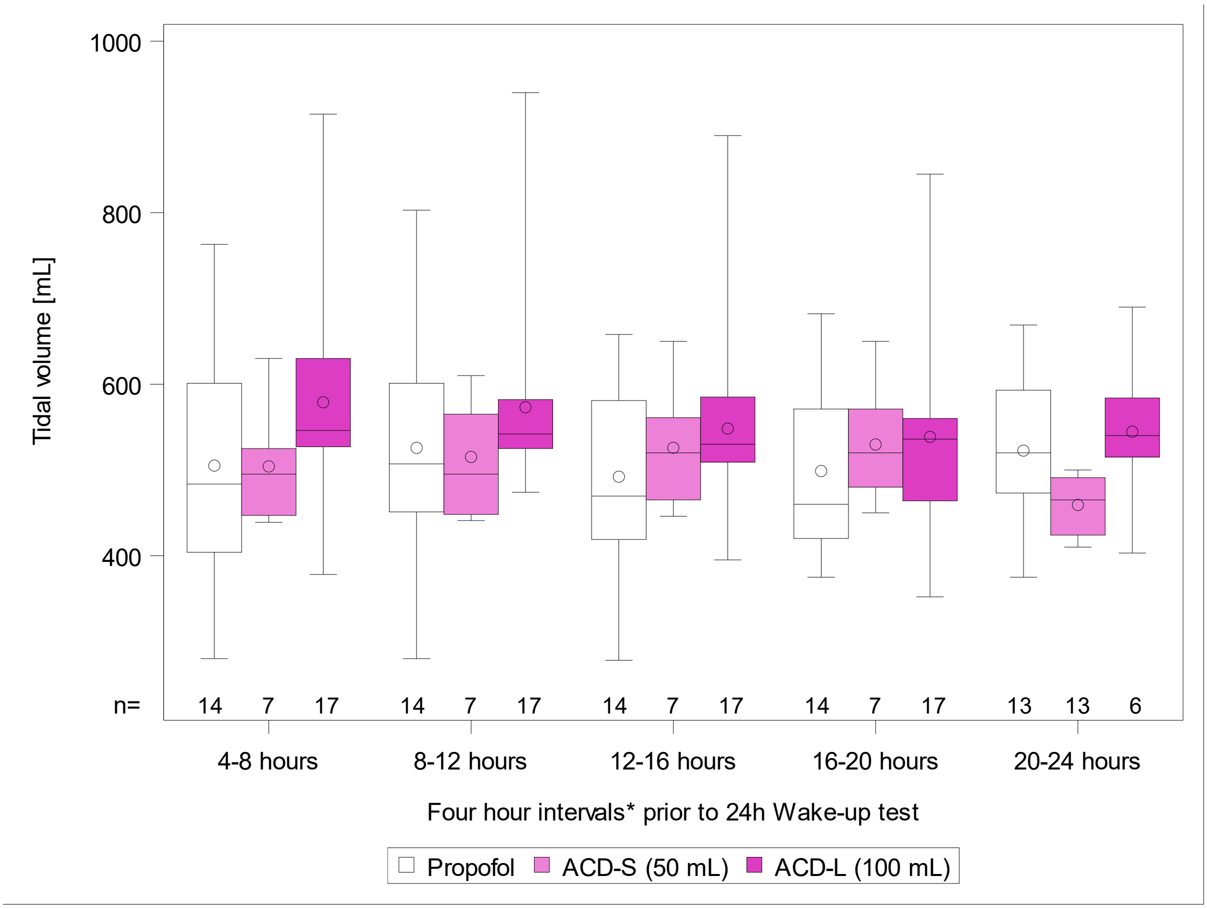

Figure S12: Tidal volume in COPD patients

* Intervals 0-4 hours and 24-28 hours have been excluded from analysis, because of the limited number of assessments available. Boxes present means, medians, and interquartile ranges. Whiskers present lowest and highest observed value within 5th percentile low and the 95th percentile high. Stars represent outliers. ACD-S, anesthetic conserving device with 50 mL internal volume. ACD-L, anesthetic conserving device with 100 mL internal volume. COPD, chronic obstructive pulmonary disease.

<u>Average differences [95% confidence interval]:</u>

ACD-L versus Propofol: 49 [-23, 121]; p=0.171

ACD-S versus Propofol: -7.4 [-105, 90]; p=0.878

ACD-L versus ACD-S: 57 [-35, 148]; p=0.216

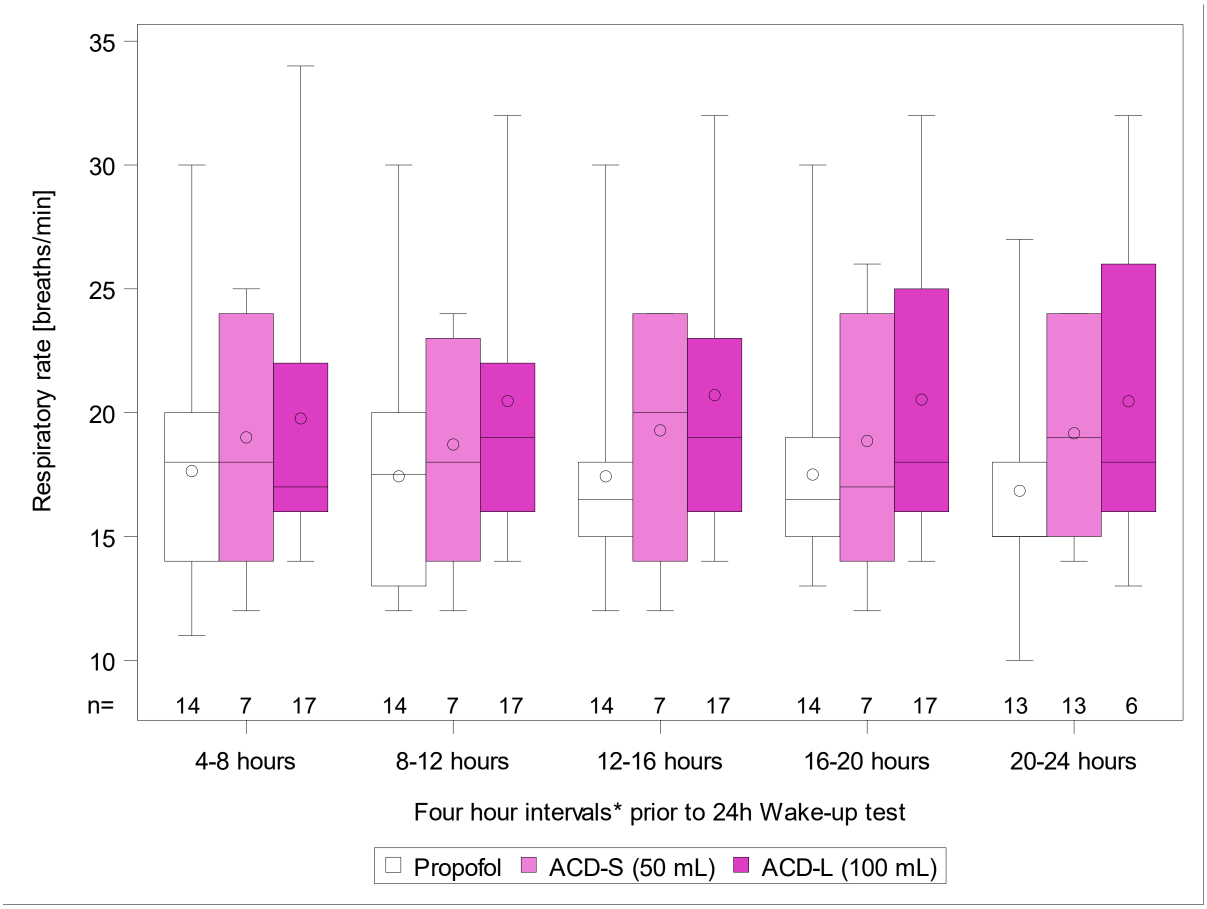

Figure S13: Respiratory rate in COPD patients

* Intervals 0-4 hours and 24-28 hours have been excluded from analysis, because of the limited number of assessments available. Boxes present means, medians, and interquartile ranges. Whiskers present lowest and highest observed value within 5th percentile low and the 95th percentile high. Stars represent outliers. ACD-S, anesthetic conserving device with 50 mL internal volume. ACD-L, anesthetic conserving device with 100 mL internal volume. COPD, chronic obstructive pulmonary disease.

<u>Average differences [95% confidence interval]:</u>

ACD-L versus Propofol: 2.5 [-0.8, 5.8]; p=0.129

ACD-S versus Propofol: 0.2 [-4.3, 4.8]; p=0.918

ACD-L versus ACD-S: 2.3 [-1.8, 6.3]; p=0.262

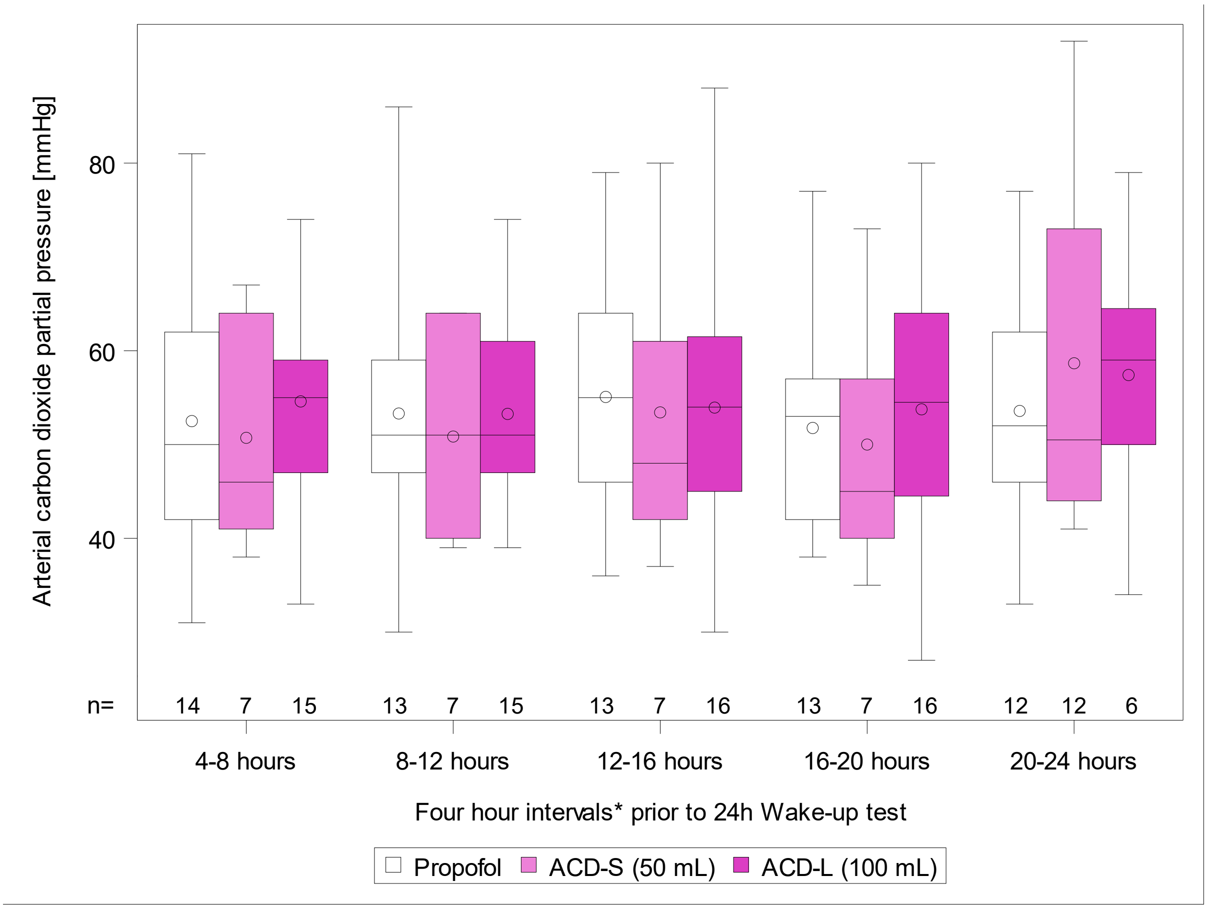

Figure S14: Arterial carbon dioxide partial pressure in COPD patients

* Intervals 0-4 hours and 24-28 hours have been excluded from analysis, because of the limited number of assessments available. Boxes present means, medians, and interquartile ranges. Whiskers present lowest and highest observed value within 5th percentile low and the 95th percentile high. Stars represent outliers. ACD-S, anesthetic conserving device with 50 mL internal volume. ACD-L, anesthetic conserving device with 100 mL internal volume. COPD, chronic obstructive pulmonary disease.

<u>Average differences [95% confidence interval]:</u>

ACD-L versus Propofol: 2.3 [-5.3, 9.9]; p=0.537

ACD-S versus Propofol: -2.1 [-12.5, 8.2]; p=0.680

ACD-L versus ACD-S: 4.4 [-4.7, 13.5]; p=0.327
